# Fungal extracellular vesicles as biomarkers in candidiasis: biogenesis, virulence, and CNS diagnostic applications

**DOI:** 10.3389/fcell.2026.1854037

**Published:** 2026-07-31

**Authors:** Jeongin Choi, Xuejin Wang, Jihyun Moon, Dae-Kyum Kim, Hunsang Lee, Ji-Eun Kim

**Affiliations:** 1 Department of Life Sciences, School of Life Sciences and Biotechnology, Korea University, Seoul, Republic of Korea; 2 National Research Laboratory for Convergence Degradation Biology, Korea University, Seoul, Republic of Korea; 3 Department of Medical and Biological Science, The Catholic University of Korea, Bucheon, Republic of Korea; 4 Cancer Research Program, The Research Institute of the McGill University Health Centre, Montreal, QC, Canada; 5 Department of Surgery, Surgical and Interventional Sciences, McGill University, Montreal, QC, Canada; 6 Department of Surgery, Division of Thoracic and Upper Gastrointestinal Surgery, Faculty of Medicine and Health Sciences, McGill University, Montreal, QC, Canada; 7 Montreal General Hospital Foundation, McGill University Health Centre, Montreal, QC, Canada

**Keywords:** biomarker, *Candida* albicans, candidiasis, extracellular vesicle, liquid biopsy

## Abstract

Invasive candidiasis remains a life-threatening infection with mortality rates reaching 40-55%, yet current gold-standard diagnostics such as blood culture are often slow and insensitive. This review evaluates fungal extracellular vesicles (EVs) as both mechanistic drivers of pathogenesis and clinical utility as biomarkers. We first outline EV biogenesis and translocation across the fungal cell wall, emphasizing the roles of turgor pressure and enzymatic remodeling. We then discuss how EVs act as protected carriers of virulence factors, such as candidalysin and Als3, which modulate tissue invasion and host responses. While profiling these circulating fungal EVs provides a promising and sensitive window into systemic invasive candidiasis, this approach faces unique hurdles in cases of central nervous system (CNS) involvement. In the CNS, fungal signatures are sequestered behind the blood-brain barrier (BBB) and heavily diluted by the vast pool of peripheral EVs, rendering total plasma analysis inadequate. To overcome these barriers, we propose a specialized diagnostic framework that combines dual-marker immunocapture of neuron-derived EVs (NDEVs), using neural cell adhesion molecule (NCAM) and intercellular adhesion molecule 5 (ICAM-5), with the detection of internal *Candida*-associated cargo. We emphasize that while this host-vesicle enrichment strategy is mechanistically grounded, it requires standardized isolation workflows and prospective clinical validation before deployment as a non-invasive platform for early CNS diagnosis.

## 1 Introduction

Fungal infections are estimated to cause up to 3.8 million deaths annually, with approximately 6.5 million invasive cases occurring worldwide each year (Denning, 2024). While several classes of potent antifungal agents are clinically available, their efficacy is increasingly compromised by escalating multidrug resistance among critical-priority pathogens, including *Candida auris* (*Candidozyma auris*), *Cryptococcus neoformans*, *Aspergillus fumigatus*, and *Candida albicans* (World Health Organization, 2022). This therapeutic challenge is further exacerbated by the inadequacies of current diagnostic tools, which often fail to identify infections early enough for effective intervention, resulting in mortality rates exceeding 40% among immunocompromised patients in intensive care units (World Health Organization, 2025).

The current diagnostic gap is particularly evident in the limitations of the gold-standard blood culture. It suffers from suboptimal sensitivity, failing to detect pathogens in up to 50% of cases, and a delayed turnaround time of 48–72 h (Fang et al., 2023). Although adjunctive non-culture assays, such as (1,3)-β-D-glucan and PCR-based panels, provide faster results, they frequently fail to distinguish active invasive infection from commensal colonization. Critically, these platforms do not translate effectively to the diagnosis of central nervous system (CNS) candidiasis, as peripheral biomarker signals are attenuated by the blood-brain barrier (BBB) (Forster et al., 2022).

Recent advances in molecular diagnostics have highlighted fungal extracellular vesicles (EVs) as informative biomarkers (Dawson et al., 2020; de Rezende et al., 2025). These lipid-bilayered nanostructures facilitate the translocation of potent virulence factors, such as the cytolytic peptide candidalysin, across the fungal cell wall to host tissues (Fang et al., 2022; Lee et al., 2025). By encapsulating proteins, lipids, and RNA (Liu and Hu, 2023), *Candida* EVs protect their cargo from host-mediated degradation and enable targeted delivery (Huang et al., 2026). Thus, profiling circulating fungal EVs offers a promising window into systemic invasive candidiasis.

However, fungal neuroinvasion, a critical yet underdiagnosed complication (Chaussade et al., 2021), presents a uniquely intractable challenge. The aforementioned BBB limits the shedding of fungal cells and biomarkers into the bloodstream, and while cerebrospinal fluid (CSF) analysis is a standard method, its invasive nature and the typically low fungal burden in the fluid often delay diagnosis (Forster et al., 2022; Samaddar et al., 2025). Furthermore, any fungal signals that do escape the CNS are heavily diluted by the vast pool of peripheral EVs in systemic circulation, limiting the sensitivity of total plasma EV analysis (Thompson et al., 2016; Mustapic et al., 2017).

To bridge the gap between basic fungal biology and its clinical application, this review details a diagnostic framework that distinguishes between *Candida*-derived EVs for systemic infection and neuron-derived EVs (NDEVs) for CNS-specific signals (Gil-Bona et al., 2015; Lee et al., 2025; Fiandaca et al., 2015). We begin by establishing the biophysical foundations of EV biogenesis and cell-wall translocation, focusing on the viscoelastic properties of the fungal cell wall (Walker et al., 2018), providing the mechanistic context required to evaluate these vesicles as high-precision biomarkers. After assessing the molecular signatures of *Candida* EVs in systemic candidiasis, we address the uniquely intractable challenge of fungal neuroinvasion, where the BBB and systemic dilution necessitate a distinct diagnostic paradigm. To overcome these barriers, we propose a specialized NDEV-based strategy utilizing dual-marker immunoaffinity capture with neural cell adhesion molecule (NCAM) and intercellular adhesion molecule 5 (ICAM-5) to isolate brain-origin fungal signatures, and we conclude by specifying the operational pipelines necessary for prospective clinical validation.

## 2 Fungal EV biogenesis and the “Great Escape”

EVs are spherical particles composed of a lipid bilayer, representing a universal mechanism of secretion across all three domains of life (Gill et al., 2019). In the field of mycology, since the first direct description in *C*. *neoformans* in 2007 (Rodrigues et al., 2007), their presence has been confirmed across at least fifteen fungal genera (Rizzo et al., 2020). While the precise biogenesis pathways of fungal EVs remain unresolved, they are presumed to follow two primary routes similar to mammalian models (Oliveira et al., 2010). The first is the microvesicle pathway, formed by direct outward budding from the plasma membrane **(** Sedgwick and D'Souza-Schorey, 2018 **)**. The second is the exosome pathway, where multivesicular bodies (MVBs) fuse with the plasma membrane to release intraluminal vesicles (Oliveira et al., 2010). Most foundational insights into the molecular machinery driving these pathways have been characterized in two representative models: the non-pathogenic yeast *Saccharomyces cerevisiae* and the opportunistic pathogen *C. neoformans*. For instance, Golgi-associated regulators, such as Sec6 in *C. neoformans* and Sec4 in *S. cerevisiae*, have been directly implicated in vesicle release and cargo sorting (Panepinto et al., 2009; Oliveira et al., 2010). In parallel, components of the endosomal sorting complex required for transport (ESCRT) machinery regulate the secretion levels of fungal EVs through MVB formation, influencing both EV cargo composition and biofilm-associated drug resistance (Zarnowski et al., 2018; Martínez-López et al., 2022).

**FIGURE 1:**
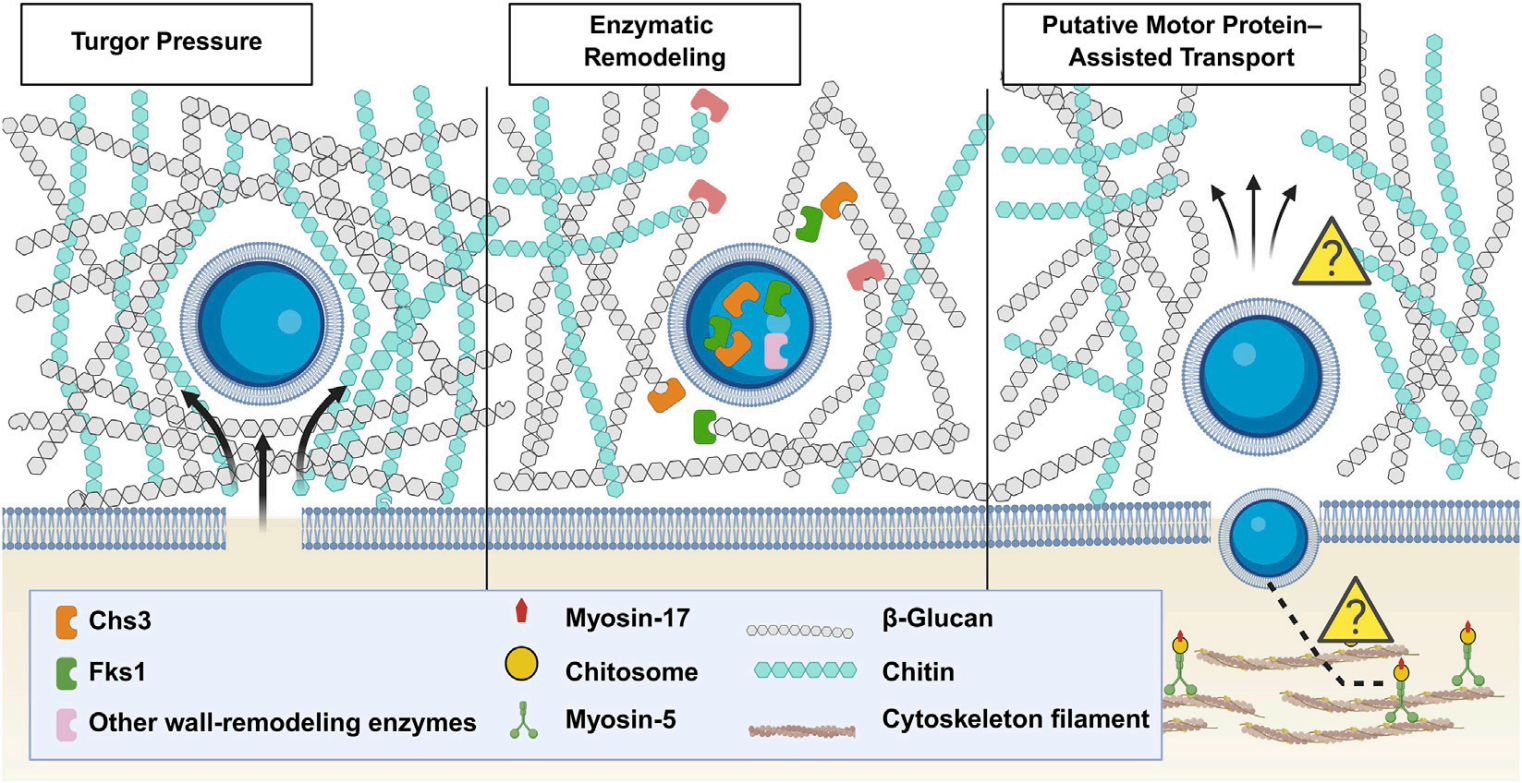
Proposed mechanisms of fungal EV translocation across the cell wall. The fungal cell wall, comprising a chitin/β-glucan network, acts as a dynamic viscoelastic barrier that membrane-bound EVs must cross to reach the extracellular space. Three non-mutually exclusive mechanisms have been proposed (Left) Turgor pressure: internal hydrostatic pressure provides the physical force to drive EVs through transient pores in the cell wall, with the viscoelasticity of the lipid bilayer permitting the deformation required for passage (Center) Enzymatic remodeling: EV-associated cell wall-remodeling enzymes locally modify the wall to facilitate transit; identified examples include the chitin synthase *Chs3* and the glucan synthase subunit *Fks1*, alongside other general remodeling enzymes (e.g., glucanases, chitinases) (Right) Putative motor protein–assisted transport: chitin synthases bearing myosin-like motor domains (Myosin-17) and myosin-related proteins (Myosin-5), potentially delivered in chitosomes, have been proposed to move EVs and chitin synthase–containing vesicles along cytoskeletal filaments toward the plasma membrane (Fernandes et al., 2016; Parreira et al., 2021; Rodrigues et al., 2025). The question mark indicates that this route remains hypothetical and awaits direct experimental confirmation. Chs3, chitin synthase 3; Fks1, 1,3-β-glucan synthase catalytic subunit.

The presence of a thick polysaccharide cell wall was previously considered a formidable physical barrier for membrane-bound EVs to exit into the extracellular space (Rizzo et al., 2020). However, recent studies have demonstrated that the fungal cell wall is not a static, rigid structure but a “viscoelastic” matrix capable of responding to external stimuli and internal turgor pressure (Walker et al., 2018). Three primary hypotheses have been proposed regarding the mechanism by which EVs traverse this barrier. First, physical force exerted by turgor pressure may push vesicles through microscopic pores within the cell wall (Wolf et al., 2014; Brown et al., 2015). Second, cell wall-remodeling enzymes associated with EVs may locally modify the cell wall structure to facilitate vesicle passage; for example, the glucan synthase subunit Fks1 and chitin synthase Chs3 have been identified as EV-enriched cargo in *S. cerevisiae*, where EV-mediated delivery of these enzymes has been linked to cell wall remodeling and antifungal resistance (Brown et al., 2015; Zhao et al., 2019). Third, motor-like proteins such as chitin synthases bearing myosin-like domains (Fernandes et al., 2016) and myosin-related proteins detected in association with EVs by proteomic approaches (Parreira et al., 2021) have been proposed to drive vesicle transit across the cell wall (Rodrigues et al., 2025) (Figure 1). This trans-wall translocation mechanism was first characterized in *C*. *neoformans* (Rodrigues et al., 2008), a process that can be metaphorically viewed as the ‘Great Escape’ of fungal macromolecules across the cell wall. It has since been extended to other pathogenic fungi, including *C*. *albicans*, where EVs similarly serve as vehicles for the delivery of virulence-associated cargo (Martínez-López et al., 2022).

## 3 EV-mediated transport of virulence factors in *Candida albicans*

**FIGURE 2:**
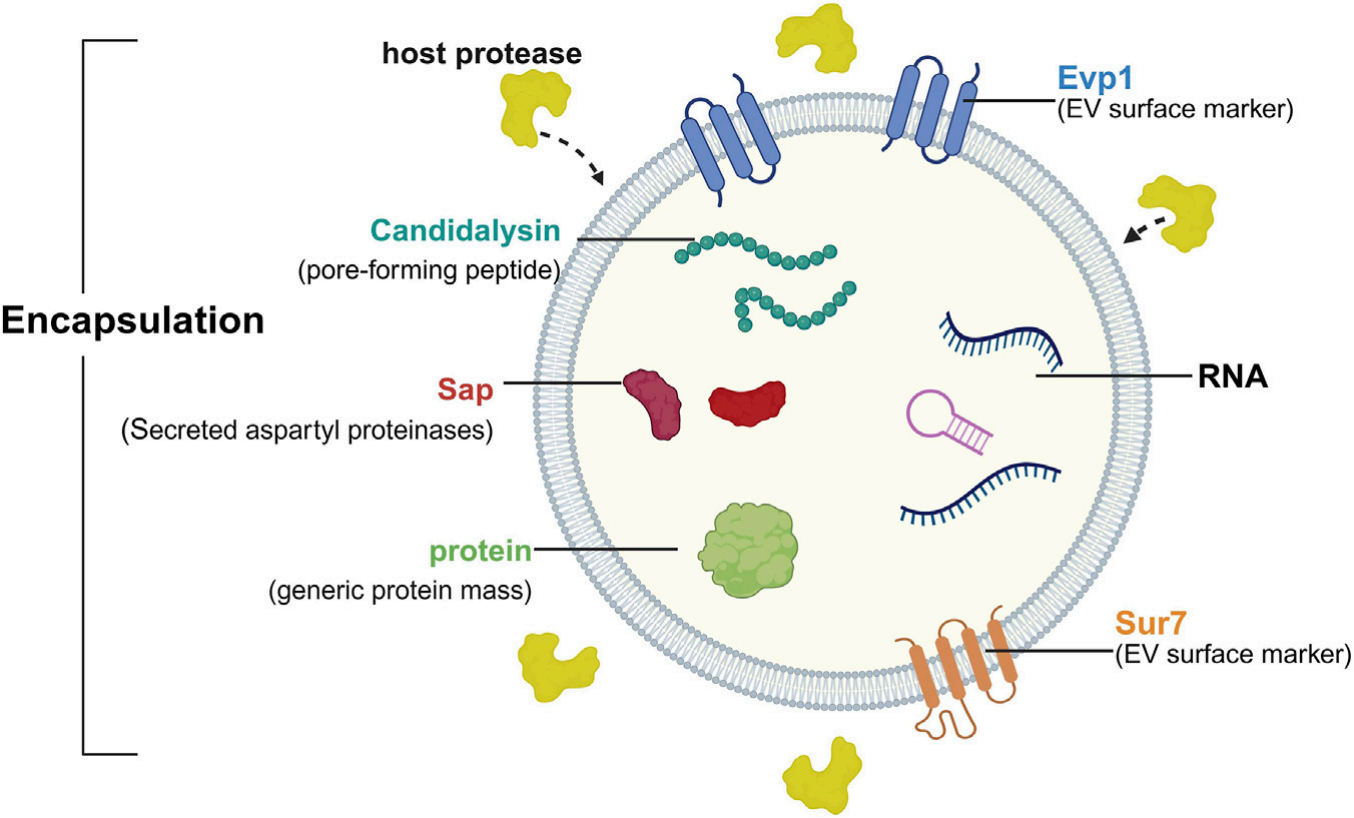
Protective encapsulation of *Candida albicans* virulence factors within extracellular vesicles. The schematic depicts a *C. albicans* extracellular vesicle (EV) in cross-section, highlighting how the lipid bilayer encapsulates virulence-associated cargo while presenting species-biased surface markers. The EV lumen contains the pore-forming peptide candidalysin, secreted aspartyl proteinases (Sap family), representative RNA species (mRNAs and small non-coding RNAs), and a generic protein mass, illustrating the molecular diversity of EV cargo. The surrounding yellow shapes represent host proteases, which are excluded by the EV membrane, emphasizing that encapsulation can shield luminal cargo from extracellular proteolysis during transit. The EV membrane is shown bearing the claudin-like proteins Sur7 and Evp1, proposed markers that are consistently enriched in *C. albicans* EVs and may support fungus-specific EV capture (Dawson et al., 2020). The molecules depicted are representative examples of EV-associated cargos and markers described in Sections 3 and 4, rather than an exhaustive inventory.

While the fundamental machinery of EV biogenesis represents a conserved mycological trait, its clinical significance is most prominently illustrated in major opportunistic pathogens such as *C. albicans*. In *C. albicans*, EVs serve as critical vehicles of pathogenesis by selectively packaging and transporting a diverse array of virulence factors that drive host cell damage (Gil-Bona et al., 2015; Vargas et al., 2015; Martínez-López et al., 2022). Among these encapsulated cargo, pore-forming toxins such as candidalysin play a central role (Lee et al., 2025). Although candidalysin is also abundantly released as a soluble toxin via the conventional secretory pathway, exerting dose-dependent cytolytic activity independently of vesicles (Moyes et al., 2016), its vesicular encapsulation serves as a sophisticated, complementary delivery mode. This targeted vehicle preserves both the immunostimulatory and membrane-permeabilizing capabilities of the peptide specifically upon direct contact with host cell surfaces (Lee et al., 2025). Furthermore, *C. albicans* EVs carry a broader proteomic signature that includes members of the secreted aspartyl proteinase (Sap) family (Martínez-López et al., 2022), which likewise function as key effectors within the extracellular milieu (Lim et al., 2021) (Figure 2).

Beyond candidalysin, the cargo repertoire of *C. albicans* EVs extends to additional effectors with distinct immunomodulatory roles. These functions encompass not only direct cytotoxicity but also the active shaping of host immune responses *in vivo*. In a murine colitis model, EVs enriched for the plasma membrane ATPase 1 (Pma1) were sufficient to aggravate intestinal inflammation, activate dendritic cells, and promote T helper 17 cell (Th17) differentiation in a caspase recruitment domain-containing protein 9 (CARD9)-dependent manner (Xu et al., 2025). These findings collectively establish *C. albicans* EVs as active modulators of host immunity whose morphology- and context-dependent cargo composition not only reflects the pathogen’s virulence state but also suggests that EV molecular profiles may serve as an informative fingerprint of infection, a property with direct implications for the diagnostic applications explored in the following section.

## 4 Diagnostic applications of fungal extracellular vesicles

The clinical management of invasive candidiasis is profoundly constrained by the inadequacies of current diagnostic tools, necessitating the development of alternative, high-precision modalities. Blood culture, the current gold standard, suffers from suboptimal sensitivity, failing to detect pathogens in up to 50% of confirmed cases, and requires a delayed turnaround time of 48–72 h (Fang et al., 2023). Although adjunctive non-culture assays, including serum (1,3)-β-D-glucan, mannan/anti-mannan antibodies, PCR panels, and the T2 magnetic resonance (T2MR)-based T2*Candida* panel, offer faster turnaround times, they share critical limitations. Specifically, these platforms frequently suffer from pan-fungal cross-reactivity, variable species-level sensitivity, and an inability to reliably distinguish active tissue invasion from benign commensal colonization (Slepčanová et al., 2026; Smadu et al., 2026). Crucially, none of these diagnostic platforms translates effectively to CNS infections, where biomarker signals are further attenuated by the BBB (Forster et al., 2022) (Table 1). It is precisely these unmet clinical needs that position circulating fungal EVs as a promising diagnostic alternative capable of providing real-time, high-specificity information (Liebana-Jordan et al., 2021; Rizzo et al., 2021).

**TABLE 1:** Comparison of current diagnostic approaches for invasive candidiasis.

Diagnostic method	Key limitation(s)	Applicability to CNS candidiasis
Blood culture (gold standard)	Low sensitivity (failing to detect pathogens in up to 50% of confirmed cases); prolonged turnaround (48–72 h); often negative in localized disease (Fang et al., 2023; Smadu et al., 2026)	Not applicable
(1,3)-β-D-Glucan	Pan-fungal, non–*Candida*-specific marker; frequent false positives (hemodialysis membranes, some antibiotics, albumin infusion); assay-dependent performance (Smadu et al., 2026; Slepčanová et al., 2026)	Requires invasive CSF sampling; serum sensitivity for CNS candidiasis is low, and CSF cutoffs not standardized (Forster et al., 2022)
Mannan/Anti-mannan antibody	*Candida*-specific but variable sensitivity across species; notably lower antigen levels in *C. parapsilosis* and *C. auris* may cause false-negative results (Smadu et al., 2026)	Not validated for CNS candidiasis (Forster et al., 2022)
*Candida* PCR panel	Variable sensitivity across species; not standardised internationally (Smadu et al., 2026)	Not routinely validated in CSF; existing CNS data are exploratory and based on small, non-standardized cohorts
T2*Candida* panel (T2MR)	Restricted to five *Candida* species; requires dedicated instrumentation; no longer widely available in many centers (Slepčanová et al., 2026)	No specific validation for CNS candidiasis; performance in CSF and in isolated CNS infection remains unknown
CSF analysis (cytology/culture)	Invasive procedure required; low fungal burden in CSF limits diagnostic sensitivity (Forster et al., 2022)	Current gold standard for CNS candidiasis diagnosis

Capitalizing on the diagnostic potential of *Candida* EVs requires a comprehensive understanding of their molecular composition, a task that has been vastly accelerated by recent advancements in high-resolution proteomics and transcriptomics. Comparative proteomic profiling has revealed that *C. albicans* EVs carry morphology-dependent virulence fingerprints whose cargo composition shifts dynamically with the pathogen’s growth state (Martínez-López et al., 2022). For instance, secreted aspartyl proteinases Sap4-6 are selectively enriched in hyphal EVs required for active organ invasion, whereas yeast-form EVs preferentially package Sap3 and the glycosylphosphatidylinositol (GPI)-anchored isoforms Sap7, Sap9, and Sap10 (Felk et al., 2002; Martínez-López et al., 2022). Hyphal vesicles are similarly enriched in other critical tissue-invasive effectors, including the adhesin Als3 and the cytolytic peptide candidalysin (Martínez-López et al., 2022; Lee et al., 2025), with EV enrichment adding to, rather than replacing, their soluble secretome-mediated delivery. These distinct proteomic profiles yield candidate biomarkers, such as Sap2, Sap4-6, Als3, and (1,3)-β-glucanosyltransferase Bgl2p (Bgl2), that report directly on the active virulence state of the pathogen (Gil-Bona et al., 2015; Martínez-López et al., 2022) (Table 2).

**TABLE 2:** Species-specific EV biomarkers and their diagnostic significance in invasive candidiasis.

Species	Marker type	Specific biomarkers	Potential clinical implication
*C. albicans*	Proteomic	^1^Sap2, ^1^Sap4-6 (hypha-enriched), ^1^Bgl2, ^1^Als3 ^2^Sur7, ^2^Evp1	^1^Linked to hyphal formation and tissue invasion; potential markers for active infection (Martínez-López et al., 2022). ^2^Identified as consistent species-specific EV enrichment markers (Dawson et al., 2020)
	Toxin	Candidalysin (Ece1-derived peptide)	EV-associated candidalysin retains pore-forming and immunostimulatory activities, serving as a candidate cargo marker for mucosal invasion (Lee et al., 2025)
*C. auris*	Transcriptomic	Species-specific RNA signatures (including tRFs and sRNA/mRNA species)	Enables culture-independent, species-level discrimination between C. auris other *Candida* species (Munhoz da Rocha et al., 2021; Zamith-Miranda et al., 2021)
*C. glabrata*	Proteomic	Epa-family GPI-anchored adhesins (e.g., Epa1)	Mediates adherence to host epithelial surfaces; the absence of secreted Sap proteinases in C. glabrata may provide a species-distinguishable EV proteomic profile compared with C. albicans (Cormack et al., 1999; Satala et al., 2025)
*C. haemulonii*	Proteomic	BMH1, TEF1, CDC19, PDC11	Linked to metabolic adaptation and intrinsic antifungal resistance; critical for stress response and niche colonization (Oliveira et al., 2025)

Abbreviations: Sap2, Sap4–Sap6, secreted aspartyl proteinases 2 and 4–6 of *C. albicans*; Bgl2, (1,3)-β-glucanosyltransferase Bgl2p of *C. albicans*; Als3, agglutinin-like sequence protein 3, hypha-associated adhesin/invasin of *C. albicans*; Sur7, Sur7 family plasma-membrane protein of *C. albicans*; Evp1, extracellular vesicle protein 1 of *C. albicans*; candidalysin, Ece1-derived cytolytic peptide toxin of *C. albicans*; Ece1, extent of cell elongation protein 1 (candidalysin parent polypeptide) of *C. albicans*; Epa1, epithelial adhesin 1, glycosylphosphatidylinositol (GPI)-anchored cell-wall adhesin of *C. glabrata*; BMH1, 14-3-3 protein Bmh1 of *S. cerevisiae*; TEF1, translation elongation factor 1-α of *S. cerevisiae*; CDC19, pyruvate kinase 1 of *S. cerevisiae*; PDC11, pyruvate decarboxylase 3 of *S. cerevisiae*.

This omics-driven mapping extends beyond proteins to the diverse RNA species encapsulated within the protected lumen of the vesicle. *Candida* EVs capture distinct transcriptomic signatures, including mRNAs and small non-coding RNAs, which are shielded from extracellular RNase degradation (Peres da Silva et al., 2015). Crucially, while tRNA-derived fragments (tRFs) are highly abundant in EVs of both species, their broader RNA profiles generate species-resolved molecular criteria that clearly differentiate the two pathogens. A key example is the significant enrichment of snoRNAs in *C. albicans* EVs, a feature that is absent in *C. auris* (Munhoz da Rocha et al., 2021; Zamith-Miranda et al., 2021). Distinctive EV signatures are also evident across other non-albicans species. Specifically, *Candida glabrata* (*Nakaseomyces glabratus*) expresses GPI-anchored Epa-family adhesins that mediate host epithelial attachment (Cormack et al., 1999) and, unlike *C. albicans*, lacks secreted Sap proteinases (Satala et al., 2025); these features may together provide a species-distinguishable EV proteomic profile. Furthermore, *Candida haemulonii* EVs transport metabolic and stress-response proteins, including *BMH1*, *TEF1*, *CDC19*, and *PDC11*, which are linked to intrinsic antifungal resistance and niche colonization (Oliveira et al., 2025). Because *C. haemulonii* is frequently misidentified as *C. auris* by standard phenotypic methods (Yadav et al., 2022), EV-based proteomic profiling among these multidrug-resistant species may have direct value for species-directed antifungal therapy. Integrating these species-specific proteomic and RNA cargos provides a dual-layer molecular architecture that can support robust, culture-independent species discrimination (Martínez-López et al., 2022; Munhoz da Rocha et al., 2021; Zamith-Miranda et al., 2021).

However, translating these highly specific molecular fingerprints into usable clinical assays introduces a substantial technical bottleneck: the efficiency of isolation and the challenge of sensitivity within a complex patient sample (Rizzo et al., 2020). Clinical specimens are dominated by an overwhelming background of host-derived vesicles, complicating the recovery of sparse fungal signals (de Rezende et al., 2025). In this context, de Rezende et al. (2025) demonstrated that EVs isolated from serum and urine of patients with candidiasis, cryptococcosis, and paracoccidioidomycosis contain metabolites of both fungal and host origin, including sterols, sphingolipids, and fatty acids. Ergosterol, a fungal-specific sterol absent in mammalian membranes, has been proposed as a potential discriminator (de Rezende et al., 2025), but its detection requires sensitive mass spectrometry-based approaches that are not yet widely accessible in clinical settings. Moreover, distinguishing true fungal EV cargo from co-isolated non-vesicular contaminants in clinical preparations remains methodologically challenging (Rodrigues et al., 2025; Rizzo et al., 2020; Welsh et al., 2024). Developing robust methods to selectively separate and enrich true fungal EVs out of the massive pool of biological noise is therefore paramount for reliable diagnostic deployment (Rodrigues et al., 2025).

To overcome this sensitivity and isolation barrier, future clinical workflows must leverage fungal-specific surface molecules to clear host background before looking at internal virulence cargos. Quantitative proteomics has identified the claudin-like Sur7 family proteins, including Sur7 and extracellular vesicle protein 1 (Evp1), which are consistently displayed on the surface of both yeast- and biofilm-derived *C. albicans* EVs, rendering them ideal targets for species-biased immunoaffinity capture (Dawson et al., 2020). In parallel, EV-delivered proton pumps like Pma1 offer a functional read-out of active mucosal infection and immune triggering *in vivo* (Xu et al., 2025). A sophisticated, two-step diagnostic platform could first isolate the *C*. *albicans* EV population using anti-Sur7/Evp1 or anti-fungal glycan antibodies, and then subsequently quantify highly specific internal cargos like Pma1 or candidalysin within this enriched fraction. If successfully implemented in clinical workflows, such a multiplexed framework would safeguard assay specificity and provide a valuable adjunct to existing serum assays, establishing the necessary precedent before confronting the even greater dilution hurdles in the CNS compartment.

## 5 Advancing CNS diagnostics: from pathogen signatures to host-derived NDEV strategies

While the isolation and profiling of circulating fungal EVs offer a promising window into systemic invasive candidiasis, this approach reaches a significant functional limit in cases involving the CNS. In systemic candidemia, fungal signatures are shed directly into the bloodstream and can be effectively captured using the fungal-specific markers (e.g., Sur7/Evp1) discussed in Section 4. However, CNS candidiasis remains a uniquely intractable diagnostic challenge because fungal signals are sequestered behind the BBB and the blood-cerebrospinal fluid barrier (BCSFB), while any signals that do escape into the periphery are heavily diluted by the vast pool of systemic EVs.

To navigate these hurdles, we must first understand the mechanisms of neuroinvasion. *Candida albicans* reaches the CNS primarily through transcellular endocytosis at the BBB, mediated by fungal invasins such as Als3 and Ssa1 (Liu et al., 2011), and via hyphal penetration at the BCSFB (Schmidt et al., 2025). Once infection is established within the brain (Schmidt et al., 2025), detecting these signals in systemic circulation becomes difficult due to the aforementioned dilution effect. Vesicles of neuronal origin constitute only a minor fraction of the total plasma EV pool, often burying CNS-origin signatures under peripheral biological noise (Mustapic et al., 2017).

Given these constraints, we propose a shift in diagnostic focus from free-floating fungal EVs to host-derived NDEVs as a superior alternative for CNS-specific signals. Although this relay strategy is not yet experimentally established in candidiasis, it is scientifically grounded in precedents from studies of other intracellular pathogens (Singh et al., 2011). In tuberculosis, for instance, host macrophages exposed to mycobacterial proteins release exosomes carrying those pathogen signatures, demonstrating that pathogen material can be internalized and re-exported within host vesicles (Giri et al., 2010). By extension, we hypothesize that fungal cargo processed by microglia (Vargas et al., 2015), the CNS-resident phagocytes, is transferred to neurons via bidirectional glial-neuronal EV exchange (Frühbeis et al., 2013; Basso and Bonetto, 2016; Ahmad et al., 2022) and subsequently shed into the blood. This relay mechanism suggests that NDEVs could serve as high-concentration capsules for CNS-origin fungal cargo that would otherwise be undetectable.

The feasibility of this future strategy is supported by the existence of established neuronal surface markers. While early reliance on L1 cell adhesion molecule (L1CAM) (Fiandaca et al., 2015) has been criticized due to its soluble, non-vesicular presence in plasma (Norman et al., 2021), a dual-marker approach using NCAM and ICAM-5 provides a promising alternative for selective NDEV enrichment. NCAM is broadly expressed across brain regions and is EV-surface-associated (Collier et al., 2025), while the telencephalic-restricted ICAM-5 provides the necessary neuron-specific precision to clear peripheral noise (Deleon et al., 2025).

**FIGURE 3:**
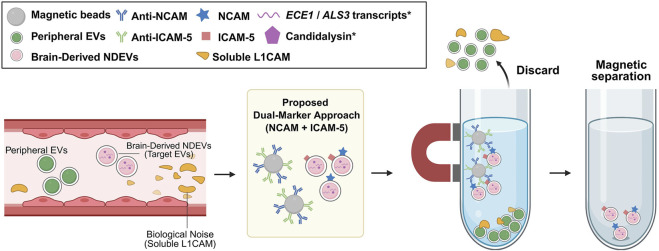
Proposed dual-marker immunocapture framework for CNS candidiasis. The schematic illustrates a strategy for enriching NDEVs from peripheral blood to concentrate otherwise dilute CNS-origin signals (Left) Systemic circulation: in blood, target NDEVs (pink) are heavily outnumbered by peripheral-tissue EVs (green) and by non-vesicular soluble L1CAM fragments (biological noise). This imbalance limits the sensitivity of unfractionated plasma EV analysis (Center) Proposed dual-marker approach (NCAM + ICAM-5): magnetic beads functionalized with both anti-NCAM and anti-ICAM-5 antibodies selectively bind NDEVs displaying NCAM and ICAM-5. This dual-marker design improves enrichment specificity over single-marker L1CAM-based approaches, which are confounded by soluble L1CAM (Right) Magnetic separation: a magnet retains the bead-bound NDEVs while unbound peripheral EVs and soluble L1CAM are discarded with the supernatant. The recovered NDEV population is proposed to harbor Candida-associated cargo, including candidalysin and *ECE1*/*ALS3* transcripts, offering a candidate non-invasive readout for CNS candidiasis. *Candidalysin and *ECE1*/*ALS3* transcripts are established cargo of *Candida*-derived EVs, but their incorporation into host NDEVs is a proposed mechanism that remains to be experimentally confirmed. NCAM, neural cell adhesion molecule; ICAM-5, intercellular adhesion molecule 5.

Ultimately, we envision that the integration of these NDEV-capture markers with the high-resolution fungal EV isolation techniques, such as the anti-Sur7/Evp1 platforms optimized in Section 4, will facilitate the recovery of candidate brain-origin signals like candidalysin or hypha-specific transcripts (*ECE1, ALS3*) from the NDEV interior, although the incorporation of these cargos into host NDEVs remains to be demonstrated experimentally. By combining host-vesicle enrichment with pathogen-specific cargo detection, this multiplexed framework offers a conceptual non-invasive platform for the early diagnosis and monitoring of CNS candidiasis (Figure 3).

## 6 Challenges in fungal EV standardization

A major barrier to translating the EV-based diagnostic framework proposed in this review into clinical practice is the lack of standardized protocols for EV isolation, characterization, and functional validation. This challenge applies directly to both classes of vesicles considered here: *Candida*-derived EVs, which underpin peripheral diagnosis (Section 4), and NDEVs, which form the basis of the proposed CNS strategy (Section 5). Unlike mammalian EV research, which has benefited from decades of methodological refinement, fungal EV studies face unique technical hurdles that complicate cross-study comparisons and limit reproducibility (Rizzo et al., 2020; Welsh et al., 2024).

Many fungal EV studies have historically relied on basic differential ultracentrifugation, a method first employed in the characterization of *C. neoformans* EVs in 2007 and since applied broadly across species (Rodrigues et al., 2007). However, this approach frequently co-isolates non-vesicular extracellular particles (NVEPs) such as protein aggregates, polysaccharide complexes, and lipoproteins, making it difficult to attribute observed biological effects to EVs alone (Zhang et al., 2018; Rizzo et al., 2020; Welsh et al., 2024). The absence of uniform reporting standards has historically led to conflicting results due to variations in culture conditions and isolation protocols, issues that the Minimal Information for Studies of Extracellular Vesicles 2023 (MISEV 2023) guidelines explicitly seek to address by requiring transparent documentation of all experimental variables (Welsh et al., 2024).

Furthermore, the fungal cell wall presents a unique physical obstacle. The dense polysaccharide matrix composed of β-glucans, mannans, and chitin can shed fragments during culture and isolation, contaminating preparations and confounding downstream proteomic and lipidomic analyses (Rodrigues et al., 2007; Rizzo et al., 2020). Moreover, the mechanisms by which EVs traverse this rigid barrier remain incompletely understood, raising questions about whether *in vitro* isolation conditions faithfully recapitulate *in vivo* vesicular release (Rodrigues et al., 2025). Additionally, unlike mammalian EVs that can be identified by well-established tetraspanin markers (CD9, CD63, CD81), universal molecular markers for fungal EVs have not yet been established, though species-specific candidates like the claudin-like Sur7 family proteins and Evp1 in *C. albicans* show promise (Dawson et al., 2020).

Pre-analytical variables such as plasma collection tubes, freeze-thaw cycles, and anticoagulant choice are also known to heavily influence EV yield and protein composition, meaning harmonized handling protocols will be mandatory prior to deployment (Welsh et al., 2024). Ultimately, the diagnostic readout proposed here requires the detection of low-abundance *Candida*-associated cargo within an already minute NDEV fraction, placing stringent demands on assay sensitivity and on the rigorous characterization of host-derived background noise. While recent fungal EV studies have begun to adopt the orthogonal characterization frameworks recommended by MISEV 2023 (Welsh et al., 2024), broader adoption remains limited, particularly in resource-constrained settings where access to cryo-EM and high-resolution mass spectrometry is lacking.

## 7 Perspectives on EV-based diagnostics and therapeutic potentials

The immediate translational priority for the framework proposed in this review lies in rigorous clinical validation. The central premise, that *Candida*-derived molecular signatures can be recovered from peripheral NDEVs, requires direct experimental confirmation using clinical specimens from patients with confirmed CNS candidiasis. Equally vital is the comprehensive benchmarking of this proposed NCAM and ICAM-5 immunocapture pipeline against established diagnostic standards, such as serum (1,3)-β-D-glucan assays, PCR panels, and CSF cultures, to determine whether it offers a genuine diagnostic advantage in terms of sensitivity and turnaround time. As these characterization workflows mature, the integration of multiplexed proteomic, transcriptomic, and lipidomic EV profiling could enable the longitudinal monitoring of pathogen virulence states in real time. In principle, this liquid biopsy framework can also be adapted to other neuroinvasive fungal pathogens, such as Cryptococcus and Aspergillus species, thereby broadening the clinical utility of host-derived EV enrichment beyond *Candida* infections.

Looking further ahead, the deepening reservoir of knowledge surrounding fungal EV biology naturally opens innovative therapeutic horizons. Interventions targeting vesicle-mediated trafficking could disrupt the delivery of potent virulence factors and alleviate biofilm-associated drug resistance. For instance, components of the ESCRT machinery and specific cell wall-remodeling enzymes might be pharmacologically targeted to impair EV biogenesis and trans-wall translocation (Zarnowski et al., 2018; Zhao et al., 2019). However, because critical toxins like candidalysin and members of the Sap family are also abundantly secreted as soluble effectors through conventional secretory pathways (Moyes et al., 2016; Lim et al., 2021), strategies targeting the EV pathway exclusively must be viewed as adjunctive modalities. Whether such vesicle-targeted interventions can achieve meaningful therapeutic efficacy without compromising host cellular viability remains an open question that demands dedicated genetic and pharmacological dissection. Ultimately, by unifying host-derived diagnostic enrichment with targeted pathogen-interdiction strategies, fungal EV biology offers a comprehensive toolkit to reshape the management of intractable invasive mycoses.

## 8 Conclusion

Fungal EVs have emerged as high-fidelity molecular messengers that bridge the gap between pathogen cell biology and clinical diagnostics. By encapsulating morphology-specific virulence factors, such as *C. albicans* hyphal Saps and *C. auris*-specific tRFs, these vesicles provide a real-time molecular readout of infection status that transcends the inherent limitations of culture-based methods. The most significant contribution of this framework is the strategic shift toward host-derived NDEVs to navigate the unique diagnostic barriers of the CNS. By bypassing the sequestration of fungal signals behind the BBB and overcoming systemic dilution through NDEV enrichment, we transform an intractable diagnostic space into a viable target for high-sensitivity liquid biopsy. While this NDEV-based paradigm is currently a hypothetical, it represents a necessary evolution in our approach to neuroinvasive candidiasis. Moving forward, the clinical success of fungal EV-based diagnostics will depend on the rigorous standardization of isolation workflows and large-scale prospective validation. Once these technical hurdles are cleared, this framework promises to redefine the management of invasive mycoses, offering a dynamic platform for early diagnosis, real-time virulence monitoring, and the eventual implementation of precision antifungal therapeutic strategies.
